# An Examination of Self-Employed Nursing Regulation in Three Canadian Provinces

**DOI:** 10.1177/15271544231175472

**Published:** 2023-05-29

**Authors:** Natalie J Thiessen, Kathleen Leslie, Jennifer M. L. Stephens

**Affiliations:** 1 Faculty of Health Disciplines, 1349 Athabasca University, Athabasca, Canada

**Keywords:** nurse's role, nurses, employment, regulation, case study, Canada

## Abstract

The COVID-19 pandemic and its related stresses such as short-staffing, heavy workloads, and burnout are prompting nurses to re-consider institutional employment, bringing a renewed interest in self-employed nursing and its regulation. There is limited research on the regulation of self-employed nursing roles, and published work focuses on nurses’ experiences rather than on regulatory practices themselves. This qualitative case study research aimed to examine the regulation of self-employed nurses by comparing the regulatory policies and processes of nursing regulatory bodies in Ontario, Alberta, and Saskatchewan. The findings demonstrated wide variation in the regulation of self-employed nurses across these jurisdictions. The article includes recommendations to clarify and harmonize the processes used to regulate self-employed nurses.

The global COVID-19 pandemic that began in 2020 exacerbated nurses’ chronic dissatisfaction with institutional nursing roles. Several Canadian member surveys conducted by nursing unions and associations in 2021 and early 2022 estimated that between 10% and 60% of nurses have considered leaving or intend to leave the nursing profession (RNAO, 2021; RPNAO, 2022; SUN, 2022) (See Table 1 for list of abbreviations). Self-employment offers nurses the opportunity to apply their unique skills, knowledge, and judgment in new and innovative self-directed roles that may improve their satisfaction and address gaps in publicly provided healthcare (Sanders & Kingma, 2012). As nurses leave institutional employment, the uptake of self-employed nursing roles may increase across Canada. It is important to nurses, provincial regulators, and the public that self-employed nursing regulation is rigorous and effective in protecting the public from harm yet does not disproportionately inhibit nurses from entering or being successful in self-employed roles. This research aimed to examine the regulation of self-employed nurses in three Canadian jurisdictions.

**Table 1.** List of Abbreviations.

Abbreviation	Explanation
CARNA	College and Association of Registered Nurses of Alberta (CRNA's previous name)
CCRNR	Canadian Council of Registered Nurse Regulators
CNA	Canadian Nurses Association
CNO	College of Nurses of Ontario
CRNA	College of Registered Nurses of Alberta
CRNS	College of Registered Nurses of Saskatchewan
LPN	Licensed Practical Nurse
NP	Nurse Practitioner
RN	Registered Nurse
RN(AAP)	Registered Nurse with Additional Authorized Practice
RNAO	Registered Nurses Association of Ontario
RPN	Registered Psychiatric Nurse
RPNAO	Registered Practical Nurses Association of Ontario
SRNA	Saskatchewan Registered Nurses Association (CRNS's previous name)
SUN	Saskatchewan Union of Nurses

## Background

A self-employed nurse practices independently, in partnership, or as an employer of others outside of the public healthcare system (CARNA, 2019a), applying their nursing knowledge, skills, and judgment in roles that enable individuals, families, groups, communities, or populations to achieve optimum levels of health (SRNA, 2021c). All Canadian nurses ([RNs], [LPNs], RPNs, and [NPs]) are overseen by provincial regulatory bodies. Provincial regulatory bodies are empowered by legislation to protect the safety and interest of the public by promoting competent practice, preventing harmful practice, and intervening in cases of misconduct or malpractice (Almost, 2021). In addition to the provincial regulator, the employer directs the practice of institutionally employed nurses through employer-specific policies and procedures. Nurses who are self-employed have more freedom to define their own policies and procedures within the legal and regulatory frameworks that apply to their profession. This increased autonomy means they are not subject to the same level of oversight as nurses who work for an employer, which allows them to practice with a broader scope of practice (CNA, 2015). Due to the increased autonomy to direct their work, the provincial regulator may perceive self-employed nurses as a higher risk to public safety. As a result, regulatory processes to protect the public interest may uniquely impact self-employed nurses. This study compared the regulation of self-employed RNs and NPs in Ontario, Alberta, and Saskatchewan.

## Review of the Literature

An extensive search for international, peer-reviewed, full-text literature on the regulation of self-employed nurses published in English between 2010 and November 2021 was conducted in five databases including Cumulative Index of Nursing and Allied Health Literature (CINAHL), ProQuest, Wiley, Ovid nursing and allied health, and Google Scholar up to the first 100 hits. Searches in each database included the following terms: entrepreneur* OR self-employ* OR private OR consult*, and nurs*, nurs* regulat*. The search for literature also included a focused but unsuccessful search of the *Journal of Nursing Regulation*. Collectively, these searches resulted in a total of 115 articles. During an assessment of titles and abstracts, 99 articles were excluded due to a lack of relevance. After a full-text review of the remaining 16 articles, only two articles reported on studies specifically related to self-employed nursing regulation: Stahlke Wall (2018) and Hunter et al. (2021).

In 2018, Stahlke Wall conducted a study using interpretive description to examine how provincial regulatory processes affect the experiences of self-employed RNs and NPs in Canada. The study involved eight participants from a single province, which was not disclosed. According to the participants, their interactions with the provincial regulator were highly negative (Stahlke Wall, 2018). The regulator was seen as imposing restrictive, unsupportive, complex, inconsistent, and unclear regulatory processes, which led to feelings of mistrust, powerlessness, and fear (Stahlke Wall, 2018). Participants in Stahlke Wall's (2018) study reported being most significantly impacted by the following: (a) the provincial regulator's operational definition of nursing practice, (b) the licensing process, (c) the peer feedback requirement associated with the quality assurance or continuing competence program, and (d) the regulatory webpage and support services. Despite being noted as negatively impacting the experiences of self-employed participants, Stahlke Wall (2018) did not examine the corresponding provincial regulatory processes to corroborate these findings.

Hunter et al. (2021) conducted a qualitative interpretive study that explored the experiences of independent nurse midwives in Australia who had been reported to the regulatory body. The study found that the regulatory investigation process had a profound emotional and psychological effect on the participants. More than half of the participants ultimately decided to exit their practice or profession, as they felt caught between their clients’ preferences and the regulatory system's limitations. Furthermore, a lack of communication and support from the regulatory body further compounded this perception, prompting the participants to leave (Hunter et al., 2021).

Stahlke Wall's (2018) study only included a small subset of self-employed nurses from a single provincial regulatory jurisdiction in Canada, which limits the generalizability of the findings. Similarly, Hunter et al.'s (2021) research focused solely on the experiences of Australian nurse midwives who had been reported to the regulatory body, thereby providing limited insights into the regulatory practices that impact self-employed nursing in Canada.

While other authors did not primarily focus on regulation, they have also documented regulatory obstacles to self-employed nursing practice in Canada (Smith, 2016; Wall, 2013a, 2013b, 2014) as well as in international settings (Cadmus et al., 2017; Colichi et al., 2019; Jahani et al., 2016; Lyden et al., 2018). These authors described regulatory barriers as a lack of knowledge of regulatory requirements, complex and lengthy licensing processes, and limitations to the scope of practice (Cadmus et al., 2017; Colichi et al., 2019; Jahani et al., 2016; Lyden et al., 2018; Smith, 2016; Wall 2013a, 2013b, 2014). This study aimed to investigate the provincial nurse regulatory processes used to regulate self-employed nurses in three Canadian jurisdictions.

## Methods

Regulatory contexts and processes vary significantly across jurisdictions; therefore this study examined and compared the regulation of self-employed RNs and NPs in three Canadian provinces. The goal was to delve beyond a surface description of regulatory content and processes to a deeper level of contextual analysis to understand the factors influencing self-employed nursing regulation. Yin's (2018) prescriptive qualitative case study methodology (see Table 2) facilitated the examination of three provincial nurse regulatory organizations, including their influential actors, contextual features, and the standards and processes used to regulate self-employed nurses.

**Table 2.** Outline of Methodological Steps and Rationale.

Steps	Related actions	Rationale and operationalization
Prestudy Tasks	Review the literature	Demonstrates knowledge of the topic and situates research. Included a broad scoping review of international literature relating to self-employed nursing and then a literature review of studies specifically focused on the regulation of self-employed nurses internationally.
	Engage in reflexivity	Protects the confirmability and dependability of the research. Maintained a reflexivity journal to investigate philosophical assumptions and personal biases throughout the research project.
Design	Define the cases	Cases chosen to accurately reflect the research question. Elected to study the regulation of RNs and NPs in various Canadian provinces to facilitate a fulsome investigation of self-employed nursing regulation.
	Select a case configuration	Appropriate arrangement of cases according to the research question allows for accurate analysis and findings. Chose three holistic cases with wide variation to allow for an effective overview of the regulation of self-employed RNs and NPs in Canada.
Selection of cases	Outline case boundaries	Cases must be clearly bounded to allow for accurate data collection and analysis. Cases included data relating to the regulation of nurses and more specifically, self-employed nursing practice.
	Complete selection process	Provides rationale for the inclusion of each case and how they are applicable to the research question. Completed a selection of cases based on several contextual factors and which of the provincial RN regulators had recently updated self-employed nursing practice guidelines.
Data collection	Identify multiple sources of data	Allows for triangulation data which is a cornerstone of case study research. Data in the form of web pages, documents, statutes, news articles, and videos was three different regulators, governments, and other public sources.
	Create a case study protocol	Outlines procedures for proceeding through the research in a systematic and rigorous fashion. Crafted a protocol that included overview of the case study, data collection procedures, guiding questions, and a tentative outline for the report.
	Establishing a study database	Stores and organizes data for future retrieval. A comprehensive database was maintained using Zotero.
	Piloting a case	Trials the case study protocol and design on one case before proceeding to the others. Small revisions to the protocol were made following the first case to further focus the data collection procedures and guiding questions.
	Maintain a chain of evidence	Demonstrates the confirmability of results. A research journal was maintained throughout the study to keep record of all decisions, notes, and memos which is stored in the database.
Data Analysis	Analysis begins with collection	Searches for initial themes and patterns. The first iteration of the analysis began when searching for data in the public realm.
	Consider data from multiple perspectives	Acknowledges different realities that may be represented within the data. Considered the perspective of the regulator, the self-employed nurse, those nurses interesting in becoming self-employed, and the public.
	Deductive and inductive approach	Build understanding of evidence from the ground up. The research question and guiding questions facilitated a deductive analysis while continually searching for emerging concepts.
	Individual case descriptions	Provides rich understanding of each case in itself. The data for each case was collected, analyzed, and reported on individually before the cross-case analysis.
	Cross-case analysis	Provides patterns and themes across cases while maintaining the integrity of each.
	Investigate all plausible rivals	Demonstrates acknowledgement of multiple realities and protects the rigor of the results. Sought alternative explanations to challenge interpretations of the data.

*Note.* Methodological steps and rationale informed by Yin (2018).

NP: Nurse Practitioner; RN: Registered Nurse.

The study was designed to include three holistic cases, each focused on a single provincial regulatory body. The study's scope did not permit an examination of the regulation of all categories of self-employed nurses, such as LPNs and nurse midwives. This limitation was due to the fact that in several provinces, these nurses are governed by distinct legislation and separate organizations from those regulating RNs and NPs. Therefore, after completing an environmental scan of all the provincial and territorial RN regulatory bodies, the College of Nurses of Ontario (CNO), the College of Registered Nurses of Alberta (CRNA), and the College of Registered Nurses of Saskatchewan (CRNS) were selected to represent the three cases included in the study. These regulatory bodies were chosen in an effort to represent maximum variation between cases based on regulatory contextual features such as (a) style of legislation, (b) whether the organization is both a regulator and a professional association, (c) the number of registrants, and (d) how recently the self-employed practice guidelines were updated (see Supplemental Appendix A). During the study, the CRNS and the CRNA split from the provincial professional associations to solely act in the public interest as a regulator which was accompanied by a re-branding in 2021 and 2022 respectively. In this text, the CRNS and the CRNA are referred to by their new titles, while the citations refer to documents published under their previous names.

The data collection phase was guided by a case study protocol which consisted of an overview of the case study, data collection procedures, guiding questions, and a tentative outline for the report (see Supplemental Appendix B). This protocol was piloted on the first case, refined, and then used to guide the data collection and analysis of the remaining cases.

Of the various provincial regulatory standards, self-employed RN and NP practice is directed most significantly by (a) the legislated and operational definition of nursing practice which outline the boundaries of the scope of practice, (b) the practice standards and guidelines directing practice within the legislated scope, and c) the guidelines specifically directing self-employed practice. As part of the licensing process, (d) declaration or currency of practice requirements, (e) recognition of nursing practice application, (f) the quality assurance or continuing competence program, and (g) the practice consultation or support services are used in the regulation of self-employed nurses. Therefore, the data collection process was made up of several systematic web-based searches to locate and download all documents and multimedia sources relevant to these specific aspects of regulation including web pages, documents, statutes, and videos from regulatory and government sources in each jurisdiction. All the data included in the study were available in the public domain and accessible on public websites. The data were loaded into a database using Zotero and then uploaded to NVivo (QSR International Pty Ltd, 2020) to facilitate further analysis. Using a deductive approach, each case was subjected to an iterative analysis, using the research questions and protocol to guide the interpretation of the data. Simultaneously, an inductive analysis facilitated the recognition of emerging concepts. After each individual case was analyzed and reported, a final, iterative cross-case analysis was completed and reported.

Credibility was strengthened by adhering to Yin's (2018) case study methods, the triangulation of data sources and the inclusion of multiple cases, using prolonged engagement with the data, reflexivity, thick description of the cases, the investigation of possible rival explanations, and the preservation of the research audit trail including the case study protocol, database, notes, memos, and research journal (Houghton et al., 2013; Squires & Dorsen, 2018). While the findings of this case study research extended to the influence of regulatory contextual features and actors on self-employed nursing regulation, this text reports the findings related to the regulatory processes used to regulate self-employed nurses in three Canadian jurisdictions.

## Findings

The CNO standards, guidelines, and processes were recently updated, were relatively clearly presented, and were not perceived to impact self-employed nurses significantly differently than those who are institutionally employed. The CRNA had very complex and unclear standards, guidelines, and processes, some of which were unique to those who are self-employed. The CRNS also had additional licensing requirements for self-employed RNs and NPs, but the standards, guidelines, and processes were recently updated and presented clearly. Table 3 presents a summary of the findings of each case.

**Table 3.** Summary of Findings Related to Regulatory Content and Processes Compared Across Jurisdictions.

Aspect of regulatory content or processes	CNO	CRNA	CRNS
Legislated and operational definition of nursing practice	- Broad legislated definition - Endorses five domains of RN practice - Includes nine roles of the RN	- Broad legislated definition - Endorses five domains of RN practice - Includes nine roles of the RN	- Broad legislated definition - Endorses five domains of RN practice - Includes nine roles of the RN
Practice standards and guidelines	- 20 total documents - Organized into subgroups - Minimal overlap between documents	- 40 total documents - Single alphabetical list - Overlapping, requires simultaneous use of multiple documents	- 22 total documents - Organized by role - Minimal overlap between documents
Self-employed practice guidelines	- Single document - Eight text pages - Option for incorporation - Unique for specifications for fees, sale of products, advertising, record keeping, and medication administration - No information on how self-employment impacts registration - Provides record of amendments - No information regarding what prompts future review	- Two checklists - Not official guideline - Inconsistent information noted on first checklist - Could be perceived to limit RN scope of practice - Lack specific expectations regarding self-employed roles - No information on how self-employment impacts registration - No publication date or information regarding what prompts future review	- Single document - 11 text pages - Describes how role fits within the practice standards and entry-level competencies - Unique for specifications pertaining to informed consent, confidentiality, information management, policy and procedure development, quality improvement and risk management, and recommendations for external consultations - No information regarding what prompts future review
Practice hours/declaration of nursing practice	- Does not require minimum number of hours - Simple process - No information regarding what constitutes “evidence of practice” when self-employed - No information about an associated audit process	- Requires 1125 minimum number of hours - Not clear on how to track self-employed hours - Not clear on associated audit process	- Requires 1125 minimum number of hours - Not clear on how to track self-employed hours - Associated audit process
Recognition of nursing practice application	- Not required	- Required - Conflicting information as to requirement to apply - No information regarding the application process, requirements, how the decision is made, time to reach a decision, or how to appeal a decision	- Required - Application requirements provided - Duplicated requirements to describe how role meets all practice standards and includes all entry-level competencies - No information regarding the time to a decision or how that decision can be appealed
Continuing competence program	- No required written peer review - Associated random audit	- Required written peer review but list of appropriate peers is inclusive - Associated audit process under review; previously directed audits on incomplete requirements of all RNs	- Required written peer review - Lack of clarity as to who qualifies as a peer - Associated random audit of all RNs
Practice consultation or support	− Response time: 1–3 days - No information regarding the process of managing consultations - No information as to the qualifications of the consultants or who manages calls from self-employed registrants	- No response time provided - No information regarding the process of managing consultations - No information as to the qualifications of the consultants or who manages calls from self-employed registrants	- Response time: 24 h - Utilizes consult management program to enhance consistency - No information as to the qualifications of the consultants or who manages calls from self-employed registrants
*Overall assessment of content and processes*	- *Simplest processes for self-employed nurses* - *Content and processes presented clearly with few exceptions*	- *Additional registration processes required for self-employed nurses* - *The least clear content and processes for self-employed registrants* - *Registrants rely fully on practice consultation to navigate registration*	- *Additional registration processes required for self-employed nurses* - *Content and processes presented clearly with a few exceptions*

CNO: College of Nurses of Ontario; CRNA: College of Registered Nurses of Alberta; CRNS: College of Registered Nurses of Saskatchewan; RN: Registered Nurse.

Overall, there were several notable similarities in all three cases. Specifically, (a) the operational definition of nursing practice, (b) the practice standards and guidelines, and (c) the quality assurance or continuing competence programs shared many commonalities across provincial jurisdictions. The most prominent distinctions among the three cases were (a) the CRNA checklists meant to guide self-employed practice, (b) the CNO's Declaration of Nursing Practice with no associated requirement for a minimum number of practice hours, and (c) the requirement for self-employed registrants of the CRNA and the CRNS to apply for recognition as a nursing practice to maintain their nursing license while practicing in self-employed roles. The individual regulatory components facilitating the regulation of self-employed nurses are presented in the following section.

### Legislated and Operational Definition of Nursing Practice

The legislated definitions of nursing practice in each of the studied jurisdictions had significant differences (see Table 4), yet the provincial regulatory bodies’ interpretations of the nursing role were almost identically broad (CARNA, 2021; CNO, 2019b; SRNA, 2015). The provincial regulatory bodies each endorsed five domains of practice, including (a) clinical, (b) education, (c) administration, (d) research, and (e) policy (CARNA, 2021; CNO, 2018a; SRNA, 2015). Each provincial regulator also recognized nurses as fulfilling nine roles, including (a) clinician, (b) professional, (c) communicator, (d) collaborator, (e) coordinator, (f) leader, (g) advocate, (h) educator, and (i) scholar (CARNA, 2019b; CNO, 2018a; SRNA, 2019a).

**Table 4.** Comparison of Legislated Definitions of Nursing Practice.

Aspect of regulatory content or processes	Definition of nursing practice
CNO	“The promotion of health and the assessment of, the provision of care for and the treatment of health conditions by supportive, preventative, therapeutic, palliative and rehabilitative means in order to attain or maintain optimal function” (Nursing Act, 1991, c. 2(3)).
CRNA	“In their practice, registered nurses do one or more of the following: (a) based on an ethic of caring and the goals and circumstances of those receiving nursing services, registered nurses apply nursing knowledge, skill and judgment to: (i) assist individuals, families, groups and communities to achieve their optimal physical, emotional, mental and spiritual health and well-being, (ii) assess, diagnose and provide treatment and interventions and make referrals, (iii) prevent or treat injury and illness, (iv) teach, counsel and advocate to enhance health and well-being, (v) co-ordinate, supervise, monitor and evaluate the provision of health services, (vi) teach nursing theory and practice, (vii) manage, administer and allocate resources related to health services, and (viii) engage in research related to health and the practice of nursing, and (b) provide restricted activities authorized by the regulations” (Health Professions Act, 2000, Schedule 24.3(a)).
CRNS	“Performance or co-ordination of health care services including but not limited to: (i) observing and assessing the health status of clients and planning, implementing and evaluating nursing care and (ii) the counselling, teaching, supervision, administration and research that is required to implement or complement health care services; for the purpose of promoting, maintaining or restoring health, preventing illness and alleviating suffering where the performance or co-ordination of those services requires: (iii) the knowledge, skill or judgment of a person who qualifies for registration” (The Registered Nurses Act, 1988, c. 2(k)).

CNO: College of Nurses of Ontario; CRNA: College of Registered Nurses of Alberta; CRNS: College of Registered Nurses of Saskatchewan.

The CRNA website directed self-employed registrants to use a checklist (CARNA, n.d.h) to determine if their service falls within the legislated and operational definition of nursing practice. Several of the CRNA's checklist items were seemingly contradictory. For example, at the top of the checklist, an opening statement suggested that all the listed items should be “checked yes” to be considered nursing practice; however, a later item on the checklist was phrased in such a way that suggests that if the item is “checked yes” it may mean the service would not be considered nursing practice. The checklist concluded with a list of roles not considered nursing practice, such as (a) those that consist primarily of activities that fall outside the nursing scope of practice (e.g., roles primarily within the scope of unregulated or other health providers), (b) focus on the sale of products, or (c) those that were “too restrictive or limited in scope” (CARNA, n.d.h, p. 2). It was unclear how these subjective evaluators were applied to determine if a specific role or service would be considered practicing nursing.

### Practice Standards and Guidelines

Practice standards published by the provincial nurse regulatory bodies had distinct similarities, as demonstrated in Table 5. The three studied provincial regulators applied the same entry-level competencies for RNs and NPs (CARNA, 2019b; CNO, 2018; SRNA, 2019a). The CRNA and the CRNS had both adopted the Canadian Nurses Association's *Code of Ethics* (CNA, 2017), and those published by the CNO (2019a) were conceptually similar. The CRNA and the CRNS also had nearly identical practice standards for RNs (CARNA, 2013; SRNA, 2019b). The CNO's practice standards were also very similar but included additional standards related to leadership and relationships (CNO, 2018c).

**Table 5.** Comparison of Practice Standards and Codes Directing Nursing Practice.

Document type	CNO	CARNA	SRNA
Code of conduct	Y	N	N
Code of ethics/ethical standards	Y	Y (CNA)	Y (CNA)
Entry-level competencies	Y (CCRNR)	Y (CCRNR)	Y (CCRNR)
Standard of practice for each nursing group	Y (NP only)	N	Y (RN, RN(AAP), NP)
Standard of practice for combined nursing groups	Y	Y	N
Scope of practice for each nursing group	N	Y (RN, NP)	N
Other practice standards	4	19	0
Total standards	9	24	5
Total practice guidelines	11	16	17
*Combined total*	*20*	*40*	*22*

*Note.* “Y” signifies yes, as the regulatory body includes a document of that type. “N” signifies no, there is not that type of document directing the practice of nurses. “Other practice standards” refers to standards pertaining to documentation, supervision, advertising, etc.

CARNA: College and Association of Registered Nurses of Alberta; CNA: Canadian Nurses Association; CNO: College of Nurses of Ontario; NP: Nurse Practitioner; RN: Registered Nurse; SRNA: Saskatchewan Registered Nurses Association.

To supplement to the core practice standards, the CNO and the CRNA published additional standards directing other aspects of practice such as documentation, privacy and health information management, advertising, and protecting patients from sexual abuse (CARNA, n.d.d; CNO, 2021d). The CNO and the CRNS websites presented practice standards and guidelines in an organized and systematic manner, facilitating their access and use (CNO, 2021d; SRNA, n.d.f). The CRNA was distinct for publishing almost twice as many practice standards and guidelines as the CNO and the CRNS and presented them in a single alphabetized list, making it challenging to identify which may apply to practice (CARNA, n.d.d). Each of the CRNA's standards and guidelines stated they were meant to be used in conjunction with several other overlapping standards and guidelines (CARNA, 2019c).

### Self-Employed or Independent Practice Guidelines

The CNO, the CRNA, and the CRNS websites provided published guidelines to assist self-employed nurses in maintaining provincial regulatory standards in this alternative role. The CNO's guideline (2021c) was unique in inviting nurses to incorporate their self-employed practice with the CNO or the Ontario government. Compared to the other two provincial nurse regulators in the study, the CNO's eight-page document included the most specifications for fees charged for nursing services, the sale of products, advertising, record keeping, and medication administration. The CNO's independent practice guidelines did not describe any additional processes nurses must engage in to be licensed as a nursing practice by the provincial regulatory body.

In contrast, the CRNA website presented self-employed registrants with a three-page checklist outlining general cautions, recommendations, and requirements related to self-employed nursing roles (CARNA, n.d.g). The checklist items appeared in point form without further direction or information. It is also unclear whether these points were recommendations or requirements.

The CRNS published an 11-page self-employed practice guideline (SRNA, 2021c) that was, by comparison, the most comprehensive. The CRNS's guideline described how the role fit within the entry-level competencies and practice standards and how self-employed nurses could be licensed by applying for recognition as nursing practice. Furthermore, the guidelines were unique in outlining specifications on informed consent, confidentiality, information management, policy and procedure development, quality improvement, and risk management. The CRNS's guidelines also provided the most detailed information about external consultations self-employed nurses should seek as they start and maintain their practice.

None of the provincial regulatory bodies’ websites or bylaws provided information on whether these guidelines were reviewed consistently or what would initiate a review. The CNO's guidelines offered a record of amendments to the document on the second page, indicating when the document was last updated (CNO, 2021c). The CRNA's checklists did not provide a date of publication or information about when or why it may be revised (CARNA, n.d.g). The CRNS's guideline did not provide a record of updates but was revised in 2021 (SRNA, 2021c). It is unclear what specifically prompted this revision.

### Declaration or Currency of Practice

These provincial regulatory bodies used specific processes to ensure registrants remain competent through recency of practice. Registrants of the CNO were not required to complete a specific number of practice hours but instead complete a Declaration of Practice on their registration renewal to report they have practiced nursing within the past 3 years (CNO, 2018b). The CNO website references “Evidence of Practice,” which seemingly describes the information that would prove the applicant has practiced nursing in the past 3 years (CNO, 2021a). However, the website did not provide information as to what qualifies specifically as “evidence” or if there was an audit process that requires the submission of that evidence.

The CRNA's and the CRNS's RN registrants were required to complete 1125 h of nursing practice within the past 5 years. NPs were required to complete 900 to 1000 h of practice within the past 3 or 4 years (Health Professions Act, 2000; SRNA, 2020). This process required nurses to track their hours and declare them on the annual registration renewal application. The CRNS established a practice hour audit process to verify the completion of the reported number of hours on registration renewal (SRNA, n.d.h). For the audit process, registrants were selected at random and required to submit proof of completed nursing practice hours (SRNA, n.d.h). There was no information about what constituted proof of nursing practice hours when nurses are self-employed. The CRNA's web page and published documents did not describe a comparable audit program of their Currency of Practice requirements (CARNA, n.d.c).

### Recognition of Nursing Practice

Self-employed registrants of the CRNA and the CRNS were required to apply for approval or recognition as a nursing practice before they may (a) use their title, (b) apply for liability protection, and (c) count their hours toward their annual registration renewal (CARNA, n.d.g; SRNA, n.d.g). The CRNA website stated self-employed nurses “may only count these hours if [their] self-employed practice has been recognized as nursing practice by CARNA” (CARNA, n.d.c, p. e1). In contrast, in a video webinar for nurses in independent practice, a presentation slide stated, “application is not needed for independent practice” (CARNA, 2020, 21:16). The website and the CRNA publications did not provide any information guiding nurses in understanding the process of gaining recognition as nursing practice, and there was no publicly accessible application form.

The CRNS website described the Recognition of Practice application, review, and decision processes (SRNA, n.d.g). Application forms were available on the website for RNs and NPs (SRNA, 2021b, 2021a). To gain recognition as a nursing practice, the self-employed CRNS registrant were required to submit (a) evidence of their registration in good standing; (b) a completed application form; (c) a written description of how the service meets the CRNS's practice standards and entry-level competencies; (d) a job description including required qualifications, responsibilities, and education; (e) proof of required education completion; and (f) additional documents and references upon request (SRNA, n.d.g, n.d.d).

The CRNS application forms required a description of (a) how the service falls within the scope of nursing, (b) how the nursing process is applied, (c) how the applicant meets and intends to maintain the required competencies, and (d) how practice standards are demonstrated in the position (SRNA, 2021b, 2021a). It was unclear if the application form also fulfilled the listed requirement to describe how the service meets the practice standards and entry-level competencies. The CRNS's self-employed practice guideline (SRNA, 2021c) highlighted a selection of practice standards and entry-level competencies that apply specifically to self-employed roles; however, the registrar required self-employed nurses to demonstrate all practice standards and entry-level competencies on the submitted application and written description (SRNA, 2021b, 2021a, n.d.g).

The CRNS website stated, “the amount of time required to render a decision is impacted by the completeness and thoroughness of the documentation submitted to support the request” (SRNA, n.d.g, p. e1). The website did not provide any further information regarding the length of time it typically takes to reach a decision. Following an initial review of the application, the CRNS's representative may provide partial or full approval, request additional information, forward the request to the Registration and Membership Committee for review, or deny the application (SRNA, n.d.g). There was no available information indicating if or how applicants can appeal the committee's decision.

### Quality Assurance or Continuing Competence Program

Provincial legislation required the CNO, the CRNA, and the CRNS to establish and maintain a program that ensures registrants maintain their competence and participate in professional development throughout their registration (Regulated Health Professions Act, 1991; Health Professions Act, 2000; The Registered Nurses Act, 1988). The Quality Assurance or Continuing Competence Programs in each jurisdiction had similar requirements, including (a) professional self-reflection, (b) peer feedback, (c) learning plan incorporating the practice standards, and (d) an evaluation of the learning plan and its impact on the registrant's nursing practice (CARNA, n.d.b; CNO, 2021b; SRNA, n.d.b).

The CNO registrants were not required to obtain written peer feedback and the CRNA's list of acceptable peers was broad (CARNA, n.d.e). The CRNS website did not explicitly define who is an acceptable colleague for peer feedback purposes. Still, various example feedback forms gave the impression that different types of written feedback from multiple sources would be acceptable (SRNA, n.d.c) although the CRNS's continuing competence program was under review at the time of the study (SRNA, n.d.a). The CNO and the CRNS websites stated registrants were randomly selected for an annual audit to verify participation in the program (SRNA, 2016). The CRNA's audit process was under review at the time of the study; however annual reports suggested they previously completed “directed” audits of registrants who submitted incomplete continuing competence requirements (CARNA, 2017, 2018).

### Practice Consultation or Support Services

All three provincial nurse regulatory bodies included in the study offered practice support by phone, mail, email, and in-person (CARNA, n.d.f; CNO, 2020; SRNA, n.d.e). The CNO and the CRNS websites publicly committed to timely responses (CNO, 2020; SRNA, n.d.e), this commitment was not publicly stated by the CRNA. There was no publicly available information on who qualifies as practice consultants or how self-employment inquiries are managed. The CRNS was unique for providing information regarding a consultation management system that stores records of practice consultations and makes them easy to retrieve, which may assist consultants in providing consistent information (SRNA, 2018, 2019a).

## Discussion

The study found that the regulation of self-employed nurses differed among the three provincial jurisdictions examined, and that the information provided on specific regulatory processes was inconsistent and unclear. This lack of consistency, clarity, and transparency may be harmful to those trying to become self-employed nurses (Stahlke Wall, 2018), and potentially undermines the public interest by delaying or preventing access to essential nursing services. Moreover, the absence of information available to the public makes it difficult to have confidence in the oversight of self-employed nursing practice. By creating consistent, transparent, and proportionate regulatory processes, and by providing detailed and accurate information about these processes, the experiences of regulated self-employed nurses can be improved, public confidence in the regulation of these nurses can be restored, and the workload of regulators may be reduced through streamlining processes. Below are the recommendations for enhancing the regulation of self-employed nursing in each case province.

The CNO did not require self-employed nurses to complete additional regulatory requirements which may facilitate such roles but may also lead to a perception of inadequate oversight. Registrants and the public in Ontario would benefit from a simple page on the CNO website that presents a clear explanation of what the self-employed role entails and how is it regulated so the information is readily available for those who may require it.

The CRNA had many overlapping practice standards and guidelines and the available information regarding self-employed nursing practice and licensure was significantly lacking, creating barriers for nurses in these roles. The CRNA website would be more effective if practice standards and guidelines were categorized rather than listed alphabetically. Additionally, a single page related to self-employed nursing practice with a description of the role, additional licensing requirements, and links to all applicable application forms would facilitate prospective self-employed registrants in navigating regulatory processes.

Like the CRNA, the CRNS had additional licensing requirements for self-employed nurses. While these additional processes were clearly described on the webpage, the application process appeared to be lengthy and had overlapping requirements. This process could potentially be significantly streamlined to be less time consuming for the registrant and the regulator.

In all cases, the regulator would benefit from tracking and reporting (a) the number of self-employed registrants, (b) the length of time recognition of practice application processes take to complete, (c) the types of self-employed roles they practice in, (d) the number and type of complaints that pertain to self-employed nurses, and (e) any practice support or consultations inquiries made by nurses in these roles. This information would provide key insight into the growth of self-employed nursing roles and any associated risk to the public which could guide the development of targeted and proportionate policies and regulation practices.

### Principle-Based or Risk-Based Regulation

All three provincial nurse regulators included in the study had adopted a form of the UK Professional Standards Authority's (PSA) risk-based, right-touch regulation focused on preventing harm by targeting practice areas of heightened risk and promoting evidence-based nursing practice (CARNA, n.d.a; CNO, 2017; PSA, 2015; SRNA, 2016). Right-touch regulation is meant to use only the means necessary to achieve desired outcomes, including protecting patients, promoting professional standards, and maintaining public confidence in the profession (PSA, 2015, 2019). Right-touch regulation is based on the principles outlined in the *Standards for Good Regulation* (PSA, 2015), which state regulation should be proportionate, consistent, targeted, transparent, accountable, and agile to the benefit of both the regulatory body, registrants, and public safety. Applying these principles to the regulation of self-employed nurses could potentially improve the clarity, transparency, and consistency of these approaches across jurisdictions while enhancing the effectiveness of regulatory processes in protecting the public.

While self-employed nurses will likely benefit from more proportionate, consistent, transparent, accountable, and agile processes, provincial regulatory bodies may choose to target self-employed nurses for additional registration or continuing competency requirements due to the more autonomous nature of their roles. Following a review of their continuing competence program, representatives of the College of Registered Nurses in Manitoba report changing their annual audit process to include 20% of NPs compared to 5% of RN registrants due to their “expanded scope of practice and the associated higher risk to the public” (Brown & Elias, 2016, p. 50), demonstrating the concept of targeting nursing roles considered to be higher risk. Despite the potential to pose additional risk, none of the provincial nurse regulators reported the number of self-employed registrants and there is no available research evaluating the risk such roles pose to the public. While it may be reasonable to target self-employed registrants with additional oversight measures due to the potential for higher risk to the public, these processes must be transparent to avoid the perception of disproportionate surveillance while providing the public confidence of rigorous regulation.

### Evidence-Informed Regulation

Although the topic of researching health practitioner regulation has been gaining traction (World Health Organization, 2021), there is a general lack of research evaluating the effectiveness of regulatory processes (Bullock et al., 2020). The CRNA and the CRNS required registrants to report a minimum number of practice hours for annual license renewal; however, the rationale supporting the specific number of required hours is unclear. Theoretically, active engagement in nursing practice should allow a nurse to maintain a degree of competence, but there is no evidence to suggest that a minimum number of practice hours is more effective than the Declaration of Nursing Practice method used by the CNO. The Recognition of Nursing Practice application processes required by the CRNA and the CRNS have also not been verified in their effectiveness in ensuring safe self-employed nursing practice.

Given the considerable impact on self-employed nurses and the additional labor, time, and regulatory resources that go into such processes, further research is required to determine their impact and effectiveness. Regulatory processes supported by research will facilitate the adoption of the same evidence-informed practices across provincial regulatory bodies, positively impacting interjurisdictional consistency.

### Limitations

Data available in the public domain lacked information regarding specific processes such as the recognition of practice application and the revision process for specific regulatory documents. Interviews with provincial regulators or access to internal regulatory documents may have supplemented the data in these areas. The CRNA and the CRNS underwent a significant change during the study, which may have impacted the availability of data and future relevancy of the findings. No two regulatory jurisdictions have identical contexts, and the impact of governing legislation, government edicts, and other contextual factors may have impacted the regulatory processes in these jurisdictions, limiting potential replicability or transferability. However, the rich description of these three jurisdictions provides a foundational analysis that may lead to practice implications for nurse regulators in other jurisdictions.

### Implications

This research builds the literature base on self-employed nursing and the regulation of unique nursing roles in Canada by demonstrating how self-employed nurses are regulated differently across provincial jurisdictions. Provincial nurse regulatory bodies should consider clarifying, augmenting, and harmonizing specific regulatory policies and processes identified in this study to facilitate the safe and effective enactment of self-employed roles. The COVID-19 pandemic and the ensuing nursing shortage has created policy and regulatory change opportunities while simultaneously demonstrating the public need for improved healthcare access (Bachtel et al., 2020; World Health Organization, 2021). Self-employed nurses should be supported with rigorous yet proportionate regulation, and the current political and regulatory environment may now support the necessary changes.

Provincial regulators may benefit from interdisciplinary collaboration with regulators of other healthcare providers that may have higher rates of self-employment such as physicians, physiotherapists, and others regarding the regulation of self-employed or independent practice roles. Such collaboration could positively impact nurse regulatory processes and improve the uptake of self-employed nursing roles.

### Future Research

Further research is required to determine the level of risk self-employed nursing roles pose to the public to better understand what targeted regulatory oversight is needed. Action research could assess the impact of principle-based regulation to determine its effect on the processes used to regulate self-employed nurses. Furthermore, the Declaration of Nursing Practice (CNO, 2018b), the requirement for a minimum number of practice hours (Health Professions Act, 2000; SRNA, 2020), and the Recognition of Nursing Practice application processes (CARNA, n.d.g; SRNA, n.d.g) should be further evaluated to determine their effectiveness in ensuring the continued competence of self-employed registrants. Other opportunities for future research include a similar study that incorporates LPNs and nurse midwives, an exploration of self-employed nurses’ perceptions of the professional and psychological impact of regulatory practices in various jurisdictions, and a comparison of self-employed regulation across different health professions.

## Conclusion

Self-employment represents a largely unexplored avenue for nurses to use their knowledge, skills, and judgment outside of institutional roles. This study demonstrates that the regulation of self-employed RNs and NPs varies significantly across the studied jurisdictions and that, in Alberta and Saskatchewan, self-employed nurses must complete additional requirements to be licensed to practice. In some cases, the information provided by the regulator regarding these additional processes is incomplete and unclear, potentially creating barriers for nurses in these roles and eroding public trust in the regulation of self-employed nursing practice. When nurses opt for self-employment, it is essential to ensure that the public has access to safe and high-quality nursing care, highlighting the need for evidence-informed regulation of independent nursing practice roles.

## Supplemental Material

sj-docx-1-ppn-10.1177_15271544231175472 - Supplemental material for An Examination of Self-Employed Nursing Regulation in Three Canadian Provinces Supplemental material, sj-docx-1-ppn-10.1177_15271544231175472 for An Examination of Self-Employed Nursing Regulation in Three Canadian Provinces by Natalie J Thiessen, Kathleen Leslie and Jennifer M. L. Stephens in Policy, Politics, & Nursing Practice

sj-docx-2-ppn-10.1177_15271544231175472 - Supplemental material for An Examination of Self-Employed Nursing Regulation in Three Canadian Provinces Supplemental material, sj-docx-2-ppn-10.1177_15271544231175472 for An Examination of Self-Employed Nursing Regulation in Three Canadian Provinces by Natalie J Thiessen, Kathleen Leslie and Jennifer M. L. Stephens in Policy, Politics, & Nursing Practice

sj-docx-4-ppn-10.1177_15271544231175472 - Supplemental material for An Examination of Self-Employed Nursing Regulation in Three Canadian Provinces Supplemental material, sj-docx-4-ppn-10.1177_15271544231175472 for An Examination of Self-Employed Nursing Regulation in Three Canadian Provinces by Natalie J Thiessen, Kathleen Leslie and Jennifer M. L. Stephens in Policy, Politics, & Nursing Practice
