# Supplementary material for: An Examination of Self-Employed Nursing Regulation in Three Canadian Provinces
Source: Policy Polit Nurs Pract. 2023 May 29;24(4):265–77. doi: 10.1177/15271544231175472 (PMC10563374; doi:10.1177/15271544231175472)
Supplement: sj-docx-1-ppn-10.1177_15271544231175472 - Supplemental material for An Examination of Self-Employed Nursing Regulation in Three Canadian Provinces [file sj-docx-1-ppn-10.1177_15271544231175472.docx]

Supplementary Material: An Examination of Self-Employed Nursing Regulation in Three Canadian Provinces

# **Appendix A**

**Case Selection Chart**

| **Province/Territory** | **Date data retrieved** | **Provincial/ territorial umbrella legislation** | **Legislated restricted activities** | **Single mandate regulatory body** | **Body regulates exclusively RNs and NPs** | **Year self-employed guidelines were last updated** |
| --- | --- | --- | --- | --- | --- | --- |
| British Columbia | April 9, 2021 | Yes  (Health Professions Act, 1996; Province of British Columbia, 2021) | Yes: “Restricted activities”  (Nurses (Registered) and Nurse Practitioners Regulation, 2008) | Yes  (British Columbia College of Nurses & Midwives, 2021a) | No: BCCNM regulates LPNs, RNs, RPNs, midwives, and NPs  (British Columbia College of Nurses & Midwives, 2021a) | Webpage - 2021  (British Columbia College of Nurses & Midwives, 2021b) |
| Alberta | April 9, 2021 | Yes  (Health Professions Act, 2000) | Yes: “Shared scope of practice/ Restricted activities”  (Registered Nurses Profession Regulation, 2005) | No: Dual mandate, but voted in 2020 to transition to single mandate  (CARNA, n.d.a) | Yes  (CARNA, n.d.a) | Webpage – 2019  (CARNA, n.d.b) |
| Saskatchewan | April 9, 2021 | No: Profession-specific act  (The Registered Nurses Act, 1988) | No | Yes: Transition from dual mandate began in 2020. No association available.  (SRNA, n.d.) | Yes  (SRNA, n.d.) | 2021  (SRNA, 2021) |
| Manitoba | April 9, 2021 | Yes  (The Regulated Health Professions Act, 2009) | Yes: “Reserved acts”  (Practice of Registered Nursing Regulation, 2017) | Yes  (College of Registered Nurses of Manitoba, n.d.-a) | Yes  (College of Registered Nurses of Manitoba, n.d.-a) | Webpage and document - n.d. (College of Registered Nurses of Manitoba, n.d.b) |
| Ontario | April 9, 2021 | Yes  (Regulated Health Professions Act, 1991) | Yes: “Authorized acts”  (Nursing Act, 1991) | Yes  (CNO, 2020) | No: RNO regulates RPNs, RNs, and NPs  (CNO, 2020) | 2021  (CNO, 2021) |
| Quebec | April 9, 2021 | Yes  (Code des professions, 1973) | Yes: “Restricted acts”  (Ordre des infirmières et infirmiers du Québec, 2019b) | Yes  (Ordre des infirmières et infirmiers du Québec, 2019c) | No: OIIQ regulates RPNs, RNs, and NPs  (Ordre des infirmières et infirmiers du Québec, 2019c) | Webpage – 2019  (Ordre des infirmières et infirmiers du Québec, 2019a) |
| Newfoundland/ Labrador | April 9, 2021 | No: Profession-specific regulation although midwives fall under separate umbrella legislation  (Registered Nurses Act, 2008, 2008; Registered Nurses Regulations, 2013) | No | Yes: Transitioned to single mandate in 2019. No association available.  (College of Registered Nurses of Newfoundland & Labrador, 2014) | Yes  (College of Registered Nurses of Newfoundland & Labrador, 2014) | 2016  (Association of Registered Nurses of Newfoundland and Labrador, 2016) |
| New Brunswick | April 9, 2021 | No: Profession-specific legislation  (Nurses Association of New Brunswick, 2002) | No | Yes: No association available.  (Nurses Association of New Brunswick, n.d.) | Yes  (Nurses Association of New Brunswick, n.d.) | 2015  (Nurses Association of New Brunswick, 2015) |
| Nova Scotia | April 9, 2021 | No: Profession-specific legislation with legislated voluntary collaboration amongst regulated groups called “The Network”  (Nursing Act, 2019; The Network, 2021) | No | Yes: Single mandate as of 2019. No association available.  (Nova Scotia College of Nursing, 2021) | No: NSCN regulates LPNs, RNs, and NPs  (Nova Scotia College of Nursing, 2021) | 2020  (Nova Scotia College of Nursing, 2020) |
| Prince Edward Island | April 9, 2021 | Yes  (Regulated Health Professions Act, 1988; Regulated Health Professions Act: General Regulations, 2018) | Yes: “Reserved activities”  (Regulated Health Professions Act: General Regulations, 2018) | Yes: Transitioned to single mandate in 2018. No association available.  (College of Registered Nurses of Prince Edward Island, 2020) | Yes  (College of Registered Nurses of Prince Edward Island, 2020) | None |
| Yukon | April 11, 2021 | No: Profession-specific act  (Yukon Registered Nurses Association, 2020) | No | No: Dual mandate  (Yukon Registered Nurses Association, 2020) | Yes  (Yukon Registered Nurses Association, 2021) | None |
| Northwest Territories/ Nunavut | April 11, 2021 | No: Profession-specific act  (Nursing Profession Act, 2004; Consolidation of Nursing Act, 1998) | No | No: Dual mandate  (Registered Nurses Association of the Northwest Territories and Nunavut, 2019a) | Yes  (Registered Nurses Association of the Northwest Territories and Nunavut, 2019a) | Webpage – 2019  (Registered Nurses Association of the Northwest Territories and Nunavut, 2019b) |

**References**

Association of Registered Nurses of Newfoundland and Labrador. (2016). *Self-employed registered nurses and nurse practitioners*. Retrieved April 11, 2021, from <https://www.crnnl.ca/sites/default/files/documents/RD_Self_Employed_Registered_Nurses_and_Nurse_Practitioners.pdf>

British Columbia College of Nurses & Midwives. (2021a). *Regulation of nurses & midwives*. British Columbia College of Nurses & Midwives (BCCNM). Retrieved April 11, 2021, from <https://www.bccnm.ca/Public/regulation/Pages/Default.aspx>

British Columbia College of Nurses & Midwives. (2021b). *Self employment*. British Columbia College of Nurses & Midwives (BCCNM). Retrieved April 11, 2021, from <https://www.bccnm.ca/NP/learning/selfemployment/Pages/Default.aspx>

*Code des Professions*, SQC 1973, c. C–26. <http://legisquebec.gouv.qc.ca/fr/document/lc/c-26>

College and Association of Registered Nurses of Alberta. (n.d.a). *About us*. College & Association of Registered Nurses of Alberta (CARNA). Retrieved April 11, 2021, from <https://nurses.ab.ca/about-us>

College and Association of Registered Nurses of Alberta. (n.d.b). *Self-employed practice*. Retrieved April 11, 2021, from <https://nurses.ab.ca/maintain-your-permit/self-employed-practice>

College of Nurses of Ontario. (2020). *What is CNO?* College of Nurses of Ontario (CNO). Retrieved April 11, 2021, from <https://www.cno.org/en/what-is-cno/>

College of Nurses of Ontario. (2021). *Practice guideline: Independent practice*. Retrieved April 11, 2021, from <https://www.cno.org/globalassets/docs/prac/41011_fsindepprac.pdf>

College of Registered Nurses of Manitoba. (n.d.-a). *About the College: What We Do*. College of Registered Nurses of Manitoba (CRNM). Retrieved April 11, 2021, from <https://www.crnm.mb.ca/about/college>

College of Registered Nurses of Manitoba. (n.d.b). *Self-Employed Practice Handbook*. Retrieved April 11, 2021, from <https://www.crnm.mb.ca/uploads/ck/files/self-employed%20handbook_jun18%20FINAL.1.pdf>

College of Registered Nurses of Newfoundland & Labrador. (2014). *Legislation and By-laws* [Text]. College of Registered Nurses of Newfoundland & Labrador (CRNNL). Retrieved April 11, 2021, from <https://www.crnnl.ca/legislation-and-laws>

College of Registered Nurses of Prince Edward Island. (2020). *About Us*. The College of Registered Nurses of Prince Edward Island (CRNPEI). Retrieved April 11, 2021, from <https://crnpei.ca/about/>

*Consolidation of Nursing Act,* SNWT 1998, c. 38, s. 4. <https://rnantnu.ca/wp-content/uploads/2019/10/Nunavut-Nursing-Act-S.N.W.T.-1998.c.38-s.4..pdf>

*Health Professions Act,* RSA 2000. c. H-7. <https://www.qp.alberta.ca/570.cfm?frm_isbn=9780779826650&search_by=link>

*Health Professions Act,* RSBC 1996, c. 183. <https://www.bclaws.gov.bc.ca/civix/document/id/complete/statreg/96183_01>

Nova Scotia College of Nursing. (2020). *Self-employed practice guideline for nurses*. Nova Scotia College of Nursing. Retrieved April 11, 2021, from <https://cdn1.nscn.ca/sites/default/files/documents/resources/SelfEmployed.pdf>

Nova Scotia College of Nursing. (2021). *Purpose, Mission, Vision & Values*. Nova Scotia College of Nursing (NSCN). Retrieved April 11, 2021, from <https://www.nscn.ca/explore-nscn/who-we-are/purpose-mission-vision-values>

Nurses Association of New Brunswick. (2002). *Nurses Act*. Nurses Assocation of New Brunswick. Retrieved April 11, 2021, from <http://www.nanb.nb.ca/media/resource/NANB-NursesAct-2008-Bilang.pdf>

Nurses Association of New Brunswick. (2015). *Guidelines for self-employed registered nurses*. Retrieved April 11, 2021, from <http://www.nanb.nb.ca/media/resource/NANB-GuidelinesSelfEmployedRNs-E.pdf>

Nurses Association of New Brunswick. (n.d.). *About NANB: Vision, Mnadate, Values & Public Protection*. Nurses Association of New Brunswick (NANB). Retrieved April 11, 2021, from <http://www.nanb.nb.ca/about/nanb>

*Nurses (Registered) and Nurse Practitioners Regulation (2020)*, BC Reg. 167/20. <https://www.bclaws.gov.bc.ca/civix/document/id/complete/statreg/284_2008>

*Nursing Act*, SNS 2019. c. 8. <https://nslegislature.ca/sites/default/files/legc/statutes/nursing.pdf>

*Nursing Act,* SO 1991, c. 32. <https://www.ontario.ca/laws/statute/91n32>

*Nursing Profession Act,* SNWT 2003, c. 15. <https://rnantnu.ca/wp-content/uploads/2019/10/Nursing-Profession-Act-S.N.W.T-2003-C.15-NPA.pdf>

Ordre des infirmières et infirmiers du Québec. (2019a). *Pratique infirmière dans le secteur privé*. Ordre Des Infirmières et Infirmiers Du Québec (OIIQ). Retrieved April 11, 2021, from <https://www.oiiq.org/pratique-professionnelle/encadrement-de-la-pratique/pratique-infirmiere-dans-le-secteur-prive>

Ordre des infirmières et infirmiers du Québec. (2019b). *Profession infirmière: Champ d’exercice et activités réservées*. Ordre Des Infirmières et Infirmiers Du Québec (OIIQ). Retrieved April 11, 2021, from <https://www.oiiq.org/pratique-professionnelle/exercice-infirmier/infirmieres-et-infirmiers>

Ordre des infirmières et infirmiers du Québec. (2019c). *Qui sommes-nous?* Ordre Des Infirmières et Infirmiers Du Québec (OIIQ). Retrieved April 11, 2021, from <https://www.oiiq.org/l-ordre/qui-sommes-nous->

*Practice of Registered Nursing Regulation (2017)*, MB Reg. 113/2017. <https://web2.gov.mb.ca/laws/regs/pre-versions.php?reg=113/2017>

Registered Nurses Profession Regulation, 232/2005 AB Reg (2005). <https://www.qp.alberta.ca/documents/Regs/2005_232.pdf>

Province of British Columbia. (2021). *Professional Regulation*. British Columbia; Province of British Columbia. Retrieved April 11, 2021, from <https://www2.gov.bc.ca/gov/content/health/practitioner-professional-resources/professional-regulation>

*Registered Nurses Act,* SNL 2008. c. R-9.1. <https://www.assembly.nl.ca/Legislation/sr/statutes/r09-1.htm>

Registered Nurses Association of the Northwest Territories and Nunavut. (2019a). *About Us*. The Registered Nurses of the Northwest Territories and Nunavut (NWTRNA). Retrieved April 11, 2021, from <https://rnantnu.mystagingwebsite.com/about/>

Registered Nurses Association of the Northwest Territories and Nunavut. (2019b). *Assessment of Nursing Practice Hours*. The Registered Nurses of the Northwest Territories and Nunavut. Retrieved April 11, 2021, from <https://rnantnu.mystagingwebsite.com/np-information/assessment-of-nursing-practice-hours/>

*Registered Nurses Profession Regulation* (2005). AB reg 232/2005. <https://www.qp.alberta.ca/1266.cfm?page=2005_232.cfm&leg_type=Regs&isbncln=9780779807123&display=html>

*Registered Nurses Regulations* (2008), NL Reg. 66/13. <https://www.assembly.nl.ca/Legislation/sr/Regulations/rc130066.htm>

*Registered Nurses Regulations* (2018). PEI Reg. EC350/18. <https://www.princeedwardisland.ca/sites/default/files/legislation/r10-1-9-regulated_health_professions_act_registered_nurses_regulations.pdf>

*Regulated Health Professions Act,* RSPEI 1988, c. R-10.1. <https://www.princeedwardisland.ca/sites/default/files/legislation/r-10-1-regulated_health_professions_act.pdf>

*Regulated Health Professions Act,* SO 1991, c. 18. <https://www.ontario.ca/laws/statute/91r18>

Saskatchewan Registered Nurses Association. (2021). *Self-Employed Practice Guidelines*. Retrieved November 7, 2020, from <https://www.srna.org/wp-content/uploads/2020/10/Self-Employed-Practice-Guidelines-RN-NP.pdf>

Saskatchewan Registered Nurses Association. (n.d.). *About the SRNA*. Saskatchewan Registered Nurses Association (SRNA). Retrieved April 11, 2021, from <https://www.srna.org/about-us/>

The Network. (2021). *The Network: Nova Scotia Regulated Health Professions Network*. NSRHPN. Retrieved April 12, 2021, from <http://www.nsrhpn.ca/>

*The Registered Nurses Act,* SS 1988, c. R-12.2. <https://www.canlii.org/en/sk/laws/stat/rss-1978-c-r-12/latest/rss-1978-c-r-12.html>

*The Regulated Health Professions Act,* SM 2009. c. R117. <https://web2.gov.mb.ca/laws/statutes/2009/c01509e.php>

Yukon Registered Nurses Association. (2020). *Yukon Registered Nurses Association Bylaws*. Yukon Registered Nurses Association (YRNA). Retrieved April 11, 2021, from <https://yukonnurses.ca/images/YRNA/Documents/Bylaws_October_2020.pdf>

Yukon Registered Nurses Association. (2021). *Home*. Retrieved April 11, 2021, from <https://yukonnurses.ca/>
