# Supplementary material for: An Examination of Self-Employed Nursing Regulation in Three Canadian Provinces
Source: Policy Polit Nurs Pract. 2023 May 29;24(4):265–77. doi: 10.1177/15271544231175472 (PMC10563374; doi:10.1177/15271544231175472)
Supplement: sj-docx-2-ppn-10.1177_15271544231175472 - Supplemental material for An Examination of Self-Employed Nursing Regulation in Three Canadian Provinces [file sj-docx-2-ppn-10.1177_15271544231175472.docx]

Supplemental Material: An Examination of Self-Employed Nursing Regulation in Three Canadian Provinces

**Appendix B**

**Case Study Protocol**

**Section A: Overview of the Case Study**

- Study Aim: This research aims to examine how self-employed nurses are regulated and which aspects of provincial nurse regulatory practices specifically impact self-employed nurses. Furthermore, this research is meant to facilitate self-employed nursing roles in Canada by supporting provincial nurse regulatory bodies and self-employed nurses in enhancing the effectiveness of self-employed nursing regulation.
- Research Question: How does provincial nurse regulation impact self-employed nurses in three Canadian provinces?

**Section B: Data Collection Procedures**

1. Data Collection Plan:

- Documentary evidence in the form of scope of practice documents, policy documents regarding self-employed nursing, informational documents, recognition of application forms, continuing competency forms, and any applicable news articles pertaining to nursing regulation in the study province
- Multimedia including webpages, presentations, videos, and social media posts published by the regulatory body and other sources available in the public domain

1. Expected Preparation Prior to Data Collection:

- Complete further research into internal organizational contextual features and adapt research design as necessary
- Create case study database
- Pilot case

**Section C: Protocol Questions**

1. Actors

- How and from whom is influence being exerted?
- What is the purpose and goals of the actors involved and how do these impact regulatory content and processes?

1. Context

- Which type of legislative framework directs nursing regulation and what aspects of regulation does it direct?
- What is the governance structure and how does it impact regulatory content and processes?
- Which regulatory philosophies direct regulatory content and processes?

1. Content

- How is nursing practice defined by legislation and the regulatory body?
- What is included in the practice standards and guidelines and how do they impact self-employed nurses?
- What is included in the guidelines for self-employed nursing practice and how do they effect these registrants?

1. Processes

- Which processes uniquely impact self-employed nurses and why?
- What resources are in place to assist nurse entrepreneurs with these processes?
- Who is involved in monitoring and evaluating the effectiveness of these processes?
- How do the regulatory processes surrounding self-employed nursing differ from that of institutionally employed nursing?

1. Impact

- How would nurses be affected by this regulatory environment?
- How would nurses perceive the processes and content that regulate their practice?
- Are there ways in which the regulation of self-employed nurses can be made more effective?

**Section D: Tentative Outline for the Case Study Report**

- Audience: Faculty of Health Disciplines at Athabasca University, nurse regulators, policymakers, nurse entrepreneurs, nursing scholars
- Individual case descriptions
- Cross-case analysis
- Findings related to external provincial context and actors and its impact on regulation
- Findings related to internal organizational context and actors and its impact on regulation
- Findings related to the impact of content and resulting processes on nurse entrepreneurs
- Suggestions for regulators and self-employed nurses
