# Supplementary material for: An Examination of Self-Employed Nursing Regulation in Three Canadian Provinces
Source: Policy Polit Nurs Pract. 2023 May 29;24(4):265–77. doi: 10.1177/15271544231175472 (PMC10563374; doi:10.1177/15271544231175472)
Supplement: sj-docx-4-ppn-10.1177_15271544231175472 - Supplemental material for An Examination of Self-Employed Nursing Regulation in Three Canadian Provinces [file sj-docx-4-ppn-10.1177_15271544231175472.docx]

**Supplemental Material Titles and Descriptions**

Appendix A – Case Selection Chart

- Description: Outlines the findings from an initial environmental scan of all Canadian provincial and territorial RN regulators which guided case selection.

Appendix B - Case Study Protocol

- Description: The data collection phase was guided by this case study protocol which consists of an overview of the case study, data collection procedures, guiding questions, and a tentative outline for the report.
